# The legal and institutional framework for the protection of religious rights in Nigeria and the right to wear Hijab in public institutions.

**DOI:** 10.12688/f1000research.132637.1

**Published:** 2023-05-23

**Authors:** CAESAR MANUCHIMSO ELIKWU, Olusola Joshua Olujobi, EBENEZER TUNDE YEBISI

**Affiliations:** 1 College of Law, Department of Public and International Law, Afe Babalola University, Ado Ekiti, Ekiti State, 360211, Nigeria; 2 College of Law, Department of Private and Business Law, Afe Babalola University, Ado Ekiti, Ekiti State, 360211, Nigeria

**Keywords:** Legal and Institutional Framework, Protection of Religious Rights, Nigeria, Hijab, Public Institutions.

## Abstract

**Background:** Religion and its exercise are one of the most sensitive discourses in different parts of the world, especially in Nigeria. The constitution and other laws ensure citizens are afforded basic human rights of which religious rights are included and yet regulated. There are questions of interpretation as the extent to which such rights can be enjoyed and the efficacy of both the legal and institutional frameworks protecting religious rights in Nigeria.

**Methods:** This article utilises a doctrinal legal research approach utilising existing literature, statutes, and laws enacted towards the protection of religious rights in Nigeria with the consideration of primary and secondary sources of laws including the 1999 Constitution of the Federal Republic of Nigeria (as amended), judicial precedents, International Conventions, law textbooks, and peer-reviewed journals. A comparative analysis of Nigeria, Tunisia, Algeria, Turkey and Kazakhstan was done to gain perspectives on balancing conflicting interests in light of Nigeria’s heterogeneous status. As part of the contribution to knowledge, a hybrid model for mitigating the socio-legal effect of the usage of hijab in Nigeria’s public institutions is presented to further enrich Nigeria’s jurisprudence.

**Results:** Nigeria, being a multi-ethnic and multi-religious state, demonstrates that the government must protect the public interests as it relates to religious rights regardless of faith or religion. The study reveals that the current legal approach without further legislative intervention on religious issues will be inadequate to address the problem. Therefore, this study presents a hybrid model for mitigating the socio-legal effect of the usage of hijab in Nigeria’s public institutions to avoid harm and further enrich Nigeria’s jurisprudence.

**Conclusions:** This study concludes that Nigeria’s legal and institutional frameworks are adequate for their purpose although they must be tweaked to conform with current trends when required to be at par with the widely accepted or world standard.

## Introduction

The wearing of hijabs in public institutions in recent times has become a discourse which has called into question the supposed secular compass of the Nigerian state following the decision of the Supreme Court in Lagos State Govt. and Ors v. Asiyat Abdulkareem, (SC/910/16), wherein the court in a split five-two judgement, held that “wearing of the hijab was an Islamic injunction and an act of worship required of Muslims and as such a fundamental right guaranteed to Muslim women” ( Premium Times, Lagos, 17 June 2022). By this decision, the Supreme had upheld the Court of Appeal’s judgement wherein it had set aside the decision of the High Court of Lagos State (Honourable Justice Coram Onyeabor) which had earlier held that the prohibition of hijabs within and outside the school premises was not discriminatory and did not go against the provisions of Sections 38 and 42 of the 1999 Constitution of the Federal Republic of Nigeria (as amended), and as such remains the current position of the law in Nigeria on hijab-wearing as a religious right.

The Supreme Court judgement has without a doubt been met with divergent views by clergymen, legal practitioners, journalists, scholars, and other members of society. For many followers of the Islamic faith, the decision of the Supreme Court upholding that of the Court of Appeal is a victory for democracy as it guarantees their rights ( Deji-Folutile, 2021).

On the other side of the divide, there is the view that the wearing of religious garbs such as the hijab in public institutions is a slippery slope that would usher in chaos as the ardent of other faiths would equally. in the light of the decision, adorn their religious attires in total disregard for the dress codes formulated by public institutions.

Nigeria being a multi-ethnic state and consequently, a multi-religious state has by this fact found itself in a peculiar position. It must find a way to protect the interests of the public as it relates to religious rights regardless of whatever faith is obtainable in whatever region in Nigeria. This article provides an exposition on the current legal and institutional framework for the preservation of religious rights as a human right in Nigeria in the context of the right to wear hijabs.

This study presents a comparative legal analysis of Tunisia, Algeria, Turkey, and Kazakhstan to that of Nigeria. The countries were selected being four advanced countries that have resolved this issue of the right to wear hijabs successfully, whereas the imbroglio of the usage of hijab in public institutions will also be considered to gain useful insight to assist Nigeria to combat this religious problem.

### Literature review

Adekola (2019) in his book contends that in light of the acrimonious nature of litigation proceedings and the strife it would result in, issues bordering on the expression of religious rights should be determined by the less confrontational methods of conciliation and mediation and other alternative disputes resolution (ADR) mechanisms. The author particularly highlights all of the instances across Nigeria where the controversy around wearing hijabs became the subject of litigation and further heated the polity. Adekola further suggests that if such controversies were brought before ADR settings a win-win approach would most likely have been adopted to assuage the concerns of all interest groups.

Adekola’s work is useful to this study as it suggests the utilization of a balanced and all-inclusive approach in addressing the hijab controversy as a human rights issue which is one of the solutions that would be explored by this study.

Ezeonu’s (2017) addresses the exasperated issue of the right to wear hijabs as a religious right within constitutional limits and the confines of public policy by appraising the Nigerian Court of Appeal (being the latest decision at the time of writing the article) decision upholding the right to wear hijabs on two major fronts. First, he lauds the judgement of the Court of Appeal in upholding the Constitutional religious rights of the citizenry as it relates to the wearing of hijabs as he notes that the said judgement is accurate in light of the failure of the Respondents (The Lagos State Government) in the instant case to plead any policy or legislation of government that prohibited the wearing of such garbs.

On the second front, Ezeonu submits that a practical way of addressing the conundrum of balancing various religious interests considering that other religious groups would seek to adorn their religious garbs, is for the government to put in place substantive legislation or at the very least guidelines and circulars stipulating a secular moral code which would prevent a descent into a state of anarchy were every faith would want to demonstrate their religious beliefs in public institutions. This he contends would not be tantamount to trampling on religious rights as such a move would be made under the spirit of the law as contained in Section 45 of the 1999 Constitution of the Federal Republic of Nigeria (as amended) empowering the state to curtail constitutional rights for public safety and order ( Ezeonu, 2017).

Ezeonu’s work provides a pragmatic approach to balancing religious interests by suggesting legislative intervention. His work is relevant to this study as it suggests legislative intervention in addressing the hijab controversy which constitutes a part of the solution this work would seek to proffer. However, the study fails to carry out a comparative legal analysis of other advanced countries that have resolved successfully the imbroglio of the usage of hijab in public institutions with useful insights Nigeria can gain to combat this religious conundrum. The gap which the current study intends to fill in the existing literature in the field of study.

Garba (2020) makes a case for the propriety of the limitation of religious rights in the country as a failure to do so would occasion anarchy in the light of the tendencies of religious groups to arbitrarily engage in activities likely to jeopardize public safety and order. He draws the instances and the need to regulate religious preaching which is a form of freedom of expression

Myland (2019) in her book review toes a similar path as Terman and Fijabi as she provides an exposition on the fact that the donning of the hijab is not necessarily an expression of the Islamic faith but has become a tool used by terror groups in propagating fundamentalist ideals of what a woman should look like. Like the work of Terman and Fijabi, the work also fails to address the views of persons who see the hijab as a true religious tenet that deserves expression.

Yusuf (2018) seeks to balance the Islamic right to wear hijabs with the duty of the government to maintain public peace and order. He submits that by the dictates of the Holy Qur’an and the Hadiths, the wearing of the hijab is a religious obligation, which a woman has to undertake. On the other hand, however, the government in the terrorism-ravaged Northeast have had reasons to place bans on hijab-wearing, in the wake of the insurgency in Northern Nigeria, as hijab-wearing has been identified as a ploy used by female suicide bombers to avoid detection. Yusuf suggests that such measures utilised by the government are truly necessary for maintaining public order and peace. Dagogo (2019), drew attention to the need for the balancing of interests when matters with religious colouration come before the government. According to them, the decision by the government in taking over missionary schools and then trying to enforce certain practices such as permitting the wearing of hijabs contributed largely to the conflicts in Osun and Kwara states as the ardent Christian faiths maintained the belief that this was an attempt by the government to trample on the sanctity of their religions.

The view expressed by Yusuf is pragmatic and indeed solidifies the claims of this study that it is appropriate to sacrifice religious practices as long as it guarantees the greater good of society.

## Methods

This article utilises a doctrinal legal research approach utilising existing literature as well as statutes and laws enacted towards the protection of religious rights in Nigeria with the consideration of primary and secondary sources of laws for instance, the 1999 Constitution of the Federal Republic of Nigeria (as amended), judicial precedents, International Conventions, law textbooks and peer-reviewed journals. Reliance will be on an examination of primary legal sources listed above. Lessons will also be drawn from international legislation and comparative analysis of Nigeria, Tunisia, Algeria, Turkey, and Kazakhstan to gain perspectives on balancing conflicting interests in light of Nigeria’s heterogeneous status. The countries were selected being advanced countries who have resolved successful the imbroglio of usage of hijab in public institutions to gain useful insight to assist Nigeria to combat this religious problem. Information on the topic of hijab restrictions in each country used for comparisons were identified using news articles without a formalised search strategy. As part of the contribution to knowledge, the study designs a hybrid model for mitigating the socio-legal effect of the usage of hijab in Nigeria’s public institutions to further enrich Nigeria’s jurisprudence.

By drawing the attention of the Nigerian populace, policy-makers, officials of the judiciary, government, and lawmakers on the need to strike an effective balance in preserving the expression of religious rights such as the wearing of hijabs in a polarised state such as Nigeria. The study emphasise the extent to which they can exercise their religious lights and civil liberties within the ambits of the law; for the judiciary it will reemphasize the need for quality judgements that cans stand the test of time in light of the doctrine of judicial precedents and its effects; and for the legislature, it shall emphasize the importance of the need for legislation on religious rights which takes into considerations the concerns of the populace in a bid to ensuring societal stability and lasting peace considering the long history of religious induced violence in Nigeria. The study further creates the necessary awareness on the various fundamental rights guaranteed under the Constitution and the need for an effective legal framework to adequately protect them. In doing so, it critically analyses the role currently played by the judiciary particularly in light of its decision in Lagos State Govt. and Ors v. Asiyat Abdulkareem, as well as the efficacy of the existing legal framework and policies for protecting religious liberties within the constitutional limits. It further appraises the ill of religious intolerance bedevilling the Nigerian society as a consequence of the government failing to manage the religious and ethnic diversity of the Nigerian peoples. Based on the comparative analysis, it makes recommendations to ensure the effective preservation of religious rights within the scope of the Constitution.

## Results

In Nigeria, a multi-ethnic and multi-religious state with various uniqueness, the federal government must protect the interests of the citizens as it relates to religious rights regardless of faith or religion. Since the current legal mechanism without further legislative intervention on religious issues is unsatisfactory to combat the scourge. As part of contribution to knowledge, the study designs a hybrid model for mitigating the socio-legal effect of the usage of hijab in Nigeria’s public institutions to further enrich Nigeria’s jurisprudence.

The article utilises a doctrinal legal research approach by utilising existing literature as well as laws enacted towards the protection of religious rights in Nigeria with the consideration of primary and secondary sources of laws for instance, the 1999 Constitution of the Federal Republic of Nigeria (as amended), judicial precedents, International Conventions, law textbooks and peer-reviewed journals. A comparative analysis of Nigeria, Tunisia, Algeria, Turkey, and Kazakhstan was done to gain perspectives on balancing conflicting religious interests in light of Nigeria’s heterogeneous status, using a utilitarian approach and law to address religious imbroglio in Nigeria.

### The legal framework for the protection of religious rights in Nigeria

The right to freedom of religion is a human right and as such an effective means to evaluate the extent of the guarantee of the right to religion is to examine the broader body of the legal framework for religious rights in Nigeria. The relevant legislations worthy of consideration in this regard include the following: The Constitution of the Federal Republic of Nigeria 1999 (as amended), the Child Rights Act 2003, and the African Charter on Human and Peoples’ Rights (Ratification and Enforcement) Act, the Universal Declaration on Human and Peoples’ Rights, 1948, the Criminal Code, and Penal Code. Others include the Federal Character Commission Act, (Establishment, Etc.) of 1996, Decree 1996 (No. 34 of 1996) and the Public Complaints Commission Act, of 1975 (No. 31) (Chapter 377). Laws of the Federation of Nigeria.

*The Constitution of the Federal Republic of Nigeria 1999* *(as amended)*

Section 38(1) of the Constitution of the Federal Republic of Nigeria 1999 (as amended), guarantees the right of Nigerian citizens to freedom of thought, conscience, and religion as follows:

“Every person shall be entitled to freedom of thought, conscience and religion, including the freedom to change his religion or belief, and freedom, (either alone or in community with others) and in public or in private, to manifest or propagates his religion or belief in worship, teaching, practice and observance.”

Upon a closer examination of the above provision, there are various aspects of the right to religious freedom beyond the broad sense which include: the right to conscience, the right to independent thought, the right to change religion, and the right to religious propagation. Indeed, in the broad sense of the right to religious freedom. Section 38(1) of the 1999 Constitution guarantees the right to adopt a religion of one’s choice. As such, every Nigerian citizen is guaranteed protection from being coerced to act in any manner adverse to his belief.

In considering religious freedom in terms of the right to conscience, a viable way the right can be exercised is by permitting a citizen to refuse compulsory military service where the same is inimical to his religious/belief system. However, this religious liberty to opt out of military service attracts a corresponding obligation under Section 34(1) of the 1999 Constitution of the Federal Republic of Nigeria which prescribes undertaking an alternative mode of service instead of military service.

A further sense in which the right to religious freedom is enjoyed under Section 38(1) of the Constitution, is the liberty afforded religious institutions to set up educational institutions which is much more articulated under Section 39(1) of the Constitution which provides that: “every person shall be entitled to freedom of expression, including the freedom to hold opinions and to receive and impart ideas and information without interference” (Section 39(1). In the case of Okogi and Others v. The Attorney General of Lagos State (1981) 2 NCLR 337, the Lagos State Government, *via* a directive, sought to abolish the operation of private primary and secondary schools to make room for the floating of its policy on education. The Plaintiffs challenged the policy because it infringed on their right to freedom of expression by preventing them from disseminating information to students. The Court declared the policy to be unconstitutional and upheld the right of religious groups to express their religious freedom by the establishment of schools to inculcate knowledge as a religious practice and even as an economic activity ( Olujobi *et al.*, 2022b).

The freedom of religion of persons attending educational institutions is another dimension in which the Constitution affords religious liberty to Nigerian citizens. Specifically, Section 38(2) of the Constitution protects the right of children to not be coerced into observing practices that are alien to their religion/that of their parents or guardians. The section reads:

“No person attending any place of education shall be required to receive religious instruction or to take part in or attend any religious ceremony or observance if such instruction ceremony or observance relates to a religion other than his own, or religion not approved by his parent or guardian” (Section 38: 2).

It is pertinent that the above section does not delineate any age limit upon which a child/individual would be able to engage in other practices alien to his religion. It is suggested in this regard that a suitable time for an individual to be able to make a such decision should be when he attains the majority age of 18 ( Onebunne & Chukwu, 2018).

Another aspect in which the right to religious freedom is exercised by Nigerian citizens is in their mode of dressing which is a subject that forms the crux of this topic. As in the Supreme Court decision, borne out of citizens approaching the courts to protect the right to wear hijabs, various individuals in other spheres of society have had the cause to do so. The cases of Miss Tulolope Ekundayo who objected to wearing trousers whilst undergoing the National Youth Service Corps (N.Y.S.C.) scheme because it offended her religious beliefs ( Afisunlu, 2013) and the continuous agitation by the Muslims Right Concern (M.U.R.I.C.) ( Lantana, 2010) are further examples of how Nigerian citizens have demanded their right to express their religious beliefs.

*The Child Rights Act 2003*

The Child Rights Act (CRA) 2003 is another legislative instrument that seeks to protect the right of Nigerian Citizens to religious freedom. The Act has been rightly described as having made more comprehensive provisions to guarantee the religious rights of children in Nigeria ( Adamu & Raji, 2020) although the opinion is also held that the CRA only accentuates the existing constitutional provisions ( Adetola-Kazeem, 2016). Through an examination of the CRA provisions on religious rights of children, this author believes however, that the act is beyond an accentuation but has provisions that can be described as more far-reaching than those of the 1999 Constitution as amended. Significantly in this regard Section 7(1), (2), (3), and (4) of the CRA read:

“(1) Every child has a right to freedom of thought, conscience and religion.

(2) Parents and, where applicable, legal guardians shall provide guidance and direction in the exercise of these rights having regard to the evolving capacities and best interest of the child.

(3) The duty of parents and, where applicable legal guardians to provide guidance and direction in the enjoyment of the right in subsection (1) of this section by their child or ward shall be respected by all persons, bodies, institutions and authorities.

(4) Whenever the fostering, custody, guardianship or adoption of a child is an issue, the right of the child to be brought up in and to practice his religion shall be a paramount consideration.”

Notably, indeed, subsections (3) and (4) beyond guaranteeing the right to religious freedom like the Constitution, emphasise the need for its respect by all entities and its protection in light of it being integral to a child’s well-being ( Intellectual Reserve Inc., 2021).

*African Charter on Human and People’s Rights (Ratification and Enforcement) Act CAP* A9, *LFN, 2004*

The African Charter on Human and People’s Rights (‘The African Charter’) equally contains relevant provisions guaranteeing the rights of Nigerians as it has been domesticated into our laws by the National Assembly. Indeed, under the domestication, courts in Nigeria are now bound to “give effect to it like all laws falling within judicial powers of the courts” (Abacha v Fawehinmi (2001) 1 CHR 20). In Abacha v. Fawehinmi, (2001) 1 CHR 20 the Supreme Court had cause to comment on the status of the African Charter *vis-à-vis* the Nigerian legal system as it notably stated that regardless of the African Charter, as an Act of the National Assembly being subservient to the Constitution, in light of its international flavour, it is superior to other Acts of the Parliament and by consequence, laws of the various Houses of Assembly.

Article 8 of the Act, like Section 38(1) of the 1999 Constitution of the Federal Republic of Nigeria (as amended) provides for the right to religious freedom and conscience as follows: “Freedom of conscience, the profession and free exercise of religion shall be guaranteed. No one may, subject to law and order, be submitted to measures restricting the exercise of these freedoms”. According to Adamu and Raji (2020), however, in contrast with the 1999 Constitution, the African Charter has no categorical provision guaranteeing the right to the private and public practice of religion or the right to change one’s religion being aspects of the right to religious freedom which the Constitution provides for. The authors submit regardless that by a holistic appreciation of the Act, these supposedly omitted senses of the right to religious freedom can be deduced.

Another way in which the African Charter expands the guarantee of the right to freedom of religion contained in the 1999 Constitution is by prohibiting religious intolerance. Article 3 guarantees equality before the law, for all individuals. Indeed, beyond the freedom from discrimination based on religion as provided by Section 42 of the Constitution, Articles 19 and 28 of the Charter prohibits religious tolerance of any form. This is a laudable provision needed in a country as pluralistic as Nigeria where without tolerance, discrimination or domination of one by the other, incidents of mistrust, conflict, and violence would be the order of the day Olujobi *et al.* (2022a).

*The Universal Declaration of Human Rights 1948*

The Universal Declaration of Human Rights (UNDHR) 1948, like the African Charter and the Constitution, guarantees the right to religious freedom. Section 18 of the Act in this regard reads:

“Everyone has the right to freedom of thought, conscience and religion; this right includes freedom to change one’s religion or belief, and freedom, either alone or in community with others and in public or private, to manifest his religion or belief in teaching, practice, worship and observance.”

The United Nations encourages member states to adopt relevant human rights provisions like the right to religious freedom and on this basis, regional legislations like the African Charter and the Constitution have been inspired to guarantee the right.

*The Criminal Code Cap.77, Laws of the Federation of Nigeria, 2004 and Penal Code Cap. 89, Laws of Northern Nigeria, 1963*

Before Nigeria gained independence in 1960, the British colonial government had enacted two separate legal regimes to function as the substantive criminal law for northern and southern Nigeria in the form of the Penal Code and the Criminal Code respectively. The Criminal Code was enacted in the year 1916 as the Nigeria Criminal Code Act 1916, while the Penal Code was enacted in the year 1960 as the Nigeria Penal Code Act 1960. Essentially, these laws purported to criminalise criminal acts and provide for the relevant punishment in a bid to discourage crime.

These legislations also play a role in preserving religious rights by providing against religious intolerance. Both legislations provide against insulting or inciting contempt of a religious creed (blasphemy) and as such Sections 204 and 210 of the Criminal Code Act and Penal Code contain punitive provisions in this regard. Section 204 of the Criminal Code in this regard reads:

“Any person who does an act which any class of persons consider as a public insult to their religion, with the intention that they should consider the act such an insult, and any person who does an unlawful act with the knowledge that any class of persons will consider it such an insult, is guilty of a misdemeanour and is liable to imprisonment for two years.” (Section 204).

Similarly, Section 210 of the Penal Code also imposes a similar imprisonment term of two years as deterrence for blasphemy. Rather unfortunately, however, the punitive provisions of these legislations have not been effectively stressed by the government as being the punishment for the crime of blasphemy. In Northern Nigeria, several examples abound of how civilians have resorted to killing persons whom they allege to have committed the offence, with the government failing to put a stop to the same as it keeps happening from time to time.

### The Institutional Framework for the Protection of Religious Rights in Nigeria

There exist several institutions/agencies of the government that through their functions serve as mechanisms for safeguarding the constitutional right to freedom of conscience and religion. These institutions include among others: The Judiciary, National Human Rights Commission, Public Complaints Commission, Truth and Reconciliation Commission, and the police.

*The Judiciary*

The Judiciary, as the third arm of government, is composed of all the courts in the country from the lowest courts, such as the Area and Customary courts to the highest Court in the land, the Supreme Court. Section 6 of the Constitution establishes Superior Courts of Record, and these include the High Courts and other courts of coordinate jurisdiction, the Court of Appeal, and the Supreme Court. Various states laws provide for courts below High Courts such as the Magistrate, Area, or Customary Courts. The role of interpreting the various statutes that govern the right to freedom of religion as well as other human rights as enshrined in the Constitution and fundamental human rights procedure rests with the judiciary. The High Court and Federal High Court as the courts of first instance in human rights matters are involved in the enforcement of fundamental human rights as defendants are tried before them with such trials sometimes leading to conviction, fines or damages as the case may be ( Ikpeze, 2013).

Indeed, the decision, which forms the crux of this article is one of the several instances in which the Courts have been called upon to address human rights issues. In *Esabunor v. Fayewa* (2019) LPELR-46961(SC). the Court of Appeal entertained the question of whether a parent could object to life-saving treatment for their child based on the former’s belief. The Court held that a mother could not object to such a lifesaving treatment thus the mother’s freedom of religion was suppressed by the interest of the state in the protection of her child’s right to live. In Medical and Dental Practitioners Tribunal v. Dr Okonkwo 7 NWLR (Pt. 711) 206 at. 237-240 however, the Court upheld the right of a patient to refuse blood transfusion on the clear basis that the decision to do so was being made by an adult of capacity electing to exercise her right to freedom of religion.

The current author submits that unlike the decision in Esabunor v. Fayewa (Supra) and Medical and Dental Practitioners Tribunal v. Dr Okonkwo (Supra *)* which took into consideration the overall public interest on the need to safeguard human rights (right to life) and what the consequence of not doing so will have on society, the decision in Lagos State Govt. and Ors v. Asiyat Abdulkareem (Supra), is too legalistic, failing to consider the pluralistic nature of Nigeria with constant suspicions of socio-political and religious domination.

*National Human Rights Commission*

The National Human Rights Commission was established by the National Human Rights Commission Act 1995 under the resolution of the United Nations enjoining her member states to set up Human Rights Institutions to guarantee and promote human rights which are inherent and universal rights to all human beings, regardless of race, sex, nationality, ethnicity, language, religion, or any other status.

The commission serves as a mechanism to enhance the enjoyment of human rights. Its establishment aims at creating an enabling environment for extra-judicial recognition, promotion and enforcement of human rights, and treaty obligations and providing a forum for public enlightenment and dialogue on human rights issues thereby limiting controversy and confrontation (Nnamani, 2011).

Under its stipulated functions under Section 5 of the Act, the commission is mandated to receive complaints of human rights violations from any person acting in his or her interest, any person acting on behalf of another person who cannot act in his or her name, any person acting as a member of or in the interest of a group or class of persons or from an association acting in the interest of its members ( Gasiokwu, 2003).

Quite commendably, the commission has handled several alleged human rights infringements across the country, including infringements on the right to religious freedom such as the suspected murder of eight people in Zamfara State on the accusation of having committed blasphemy ( Chude, 2023) This current author, however, submits that a lot of work is still left undone by the Commission particularly as it relates to the preservation of the right to religious freedom particularly when one considers the spate of killings fuelled by religious intolerance, particularly in Northern Nigeria. In recent memory is the case of Deborah Samuel, a student at a College of Education in Sokoto State, beaten to death and set ablaze by her irate colleagues on the grounds of blasphemy. Considering such gruesome scenarios continue to play out, one is left to wonder how effective the Commission has been in effecting its mandate on human rights protection in Nigeria.

*Federal Character Commission*

Section 153 of the 1999 Constitution of the Federal Republic of Nigeria obligates the Federal Government to establish a Federal Character Commission. Accordingly, the Federal Character Commission is mandated by its enabling Act “to promote, monitor and enforce compliance with the principles of the proportional sharing of all bureaucratic, economic, media and political posts at all levels of governments” as stated in the preamble to the Federal Character (Establishment) Act, 2004.

Owing to Nigeria’s diverse nature in all aspects, the role of the Commission is particularly important in allaying fears of ethnic and more particularly religious domination. As such by ensuring that economic, social and political opportunities in the country are evenly distributed regardless of an individual’s religious affiliation, the right to express one’s religious beliefs is guaranteed as citizens will do so without the fear of victimization.

The Commission has however come under severe criticism because it has failed to guarantee an even spread of job opportunities and it sacrifices excellence on the altar of mediocrity by allowing for the employment of persons or admission of students based on filling ‘quota’ systems meant for regions of the country.

*Public Complaints Commission*

The Public Complaint Commission is established by the Public Complaints Commission Act 1975. By Section 5 of the Act, the Commission is given an array of powers to inquire into complaints by members of the public about the administrative action of any public authority and companies or their officials and other related matters.

To ensure the protection of human rights and prevention of injustice, the Commission is mandated to ensure that public officials or institutions do not carry out any act of injustice against any citizen of Nigeria or any other person resident in Nigeria and for that purpose, the commission shall investigate with special care administrative acts which appear to be contrary to any law or regulation; mistaken in law or arbitrary in the ascertainment of fact; unreasonable, unfair, oppressive or inconsistent with the general functions of administrative organs; improper in motivation or based on irrelevant considerations; unclear or inadequately explained, or otherwise objectionable under Section 5(3) of the Public Complaint Commission Act, 1975.

Section 8(3) imposes a meagre fine of N500 or six months imprisonment on any person who obstructs/induces/interferes with an officer of the Commission in the performance of his duty and Section 8(4) imposes a jail term of one-year imprisonment without the option of a fine on any person who, upon being called upon by the Commission in the course of an investigation, makes any false statement. This writer submits that the punitive mechanism currently put in place by the Commission is insufficient to deter would-be offenders. Worthy of note is that at the time of this research, the current author is unaware of any convictions made under the Act, and this is perhaps suggestive of the lacklustre disposition of the Commission in carrying out its mandate.

*Truth and Reconciliation Commission*

The Truth and Reconciliation Commission widely known as the Oputa Panel was an Ad hoc Commission established in the wake of the collapse of decades of military government in Nigeria. Setup by former President Olusegun Obasanjo, the mandate of the Commission was to inquire into human rights abuses between 1984 to 199 ( Hayner, 2010). Although the Commission forwarded its findings to the President in 2002, no major action has been implemented regarding the report submitted to date. Commendably, however, the Commission is known to have helped reconcile Nigerian communities in conflict such as several Ogoni communities’ issues.

*The police and other security agencies*

The Police and other law enforcement agencies of the state are established and governed by their specific enabling laws. The Police Act for instance empowers the Police to investigate and arrest persons for offences under any extant law and by implication, certain human rights infringements such as murder for instance, Sections 4 and 23 of Police Act Cap P19, LFN 2020 subject only to the constitutional power of the Attorneys General under the Constitution (This is the power of nolle prosequi granted under Sections 174 and 211 of the Constitution to the Attorney General of the Federation and Attorney General of the State respectively). The National Securities Agencies Act provides for three agencies namely: The Defence Intelligence Agency (DIA); the National Intelligence Agency (NIA); and the State Security Service (SSS). The SSS is the most visible among the trio. Although the statute tried to delineate their functions, they practically dovetail, interrelate or integrate sometimes to assist Nigeria to combat this religious problem ( Ikpeze, 2013).

### Comparison with other countries

The results of the comparisons are detailed below with each of the selected countries and sources used for each country in the point-by-point comparative analysis of how each country resolved the imbroglio of wearing of hijabs in public institutions. Information on the topic of hijab restrictions were identified using news articles for each country without a formalised search strategy. The countries banned hijabs in public institutions due to security threats and to combat religious radicalism.

*Nigeria*

The wearing of the female Islamic dress called hijab is regarded amongst a majority of Muslim faithful as a mandatory religious injunction which has generated complex arguments particularly in the context of human rights to freedom of religion and non-discrimination in education and employment. Respect for individual Muslim women to wear or not wear hijab is fundamental to curtail infringement of their Constitutional rights. This was upheld by the Supreme Court in Lagos State Govt. and Ors v. Asiyat Abdulkareem. However, the Supreme Court decision is now widely regarded as an invitation to chaos in light of the highly pluralistic nature of the Nigeria state and its supposed secular constitution. The case has opened the floodgate of chaotic interpretations which require legislative intervention to address the problem. Nigeria should emulate states like Turkey, regardless of being an Islamic nation, have entrenched secular constitution to allay any internal or concerns from the international community that objective fairness devoid of religious sentiment is not being practiced in the country. Indeed, state resources should be channelled towards the promotion of education, healthcare, industrialisation, the overall wellness of the economy, and not private matters like religion. Nigeria must arrive at a solution in interpreting/managing the decision reached by the Supreme Court in Lagos State Govt. and Ors v. Asiyat Abdulkareem (Supra), in a manner that does not result to carnage.

*Tunisia*

Tunisia, with a significantly higher Muslim population than Nigeria, has banned the use of hijabs in public institutions such as universities. Owing to its status as a secular state, the Tunisian government has a ban in force against the wearing of hijabs in public institutions such as schools and workplaces, with the aim of curbing the threats posed by the activities of Islamic fundamentalists ( Hawkins, 2011). The view has also been expressed that the ban is also part of the nation’s attempts at gaining global acceptance as a non-extremist state ( Simon, 2011). Tunisia, who has resolved successful the imbroglio of usage of hijab in public institutions, will also be considered to gain useful insight to assist Nigeria to combat this religious problem by imbibing global best practices geared towards improving the framework for the preservation of constitutional rights in Nigeria. Tunisia and Turkey are two countries who have decided against its wearing in public places.

*Algeria*

Before the ban on public and civil service institutions, Algeria’s Education Ministry had also imposed a ban on hijabs because it also encouraged cheating and posed a security threat ( Middle East Online, 2022). Like Nigeria, where the wearing of hijabs in public schools resulted in clashes between Muslims and Christians, the said ban on hijabs in Algeria resulted in confrontations between the majority Sunni Muslims in support of the ban and the significantly sized Salafist population who is more conservative in their practice of Islam opposed the ban ( Rahim, 2022).

*Turkey*

Although Turkey operates as a secular state, it has a majorly Muslim population. Hijabs are not prohibited in the country, rather the country promotes their usage as a symbol of its religious heritage ( Reuters, 2022). Between the 1920s and the 2000s, owing to European influence, Turkey had previously placed a ban on hijabs and other headscarves as part of its show of secularism as a European state. With a change of government in the early 2000s, the state lifted the ban on headscarves in various public institutions including judiciary and military ( Handy, 2022).

*Kazakhstan*

Like its Algerian counterpart, Kazakhstan banned the use of hijabs in public schools in the year 2016 through a directive issued by the nation’s education Ministry ( Urnaliev & Najibullah, 2022). The ban is informed by the concerns of the state that religious extremists who utilize such clothing to occasion harm in this society.

## Discussion

The current legal framework for the protection of human rights in Nigeria inclusive of religious rights is composed of a plethora of laws as well as institutions saddled with the responsibility of guaranteeing various human rights such as the right to wear hijab in public institutions as a religious right. This makes the role of the judiciary very critical as it is invariably responsible for giving effects to the letters of the law upon which persons who seek enforcement of their human rights can bring before it. This study has shown that although Nigeria is not bereft of working institutions like the judiciary, and legislation to address human rights issues such as the wearing of hijabs, the decision of the Supreme Court in Lagos State Govt. and Ors v. Asiyat Abdulkareem (Supra), which has seemingly opened a floodgate of chaotic interpretations, has shown that the current legal approach without further legislative intervention or a hybrid of social and legal solutions will be inadequate to address the problem.

The study has also found that to effectively address the plausible chaotic aftermath of the decision in Lagos State Govt. and Ors v. Asiyat Abdulkareem (Supra) as it relates to human rights, the government must take steps to promote a balanced and fair state where heterogeneous and pluralistic factors are well considered, by genuinely giving credence to the provisions of the constitution that project Nigeria as a secular state.

This study also brings to fore the reality that for any guarantee of human rights in the country to be sustained, there must be sincerity of purpose and the right politics should be shown by the government to tackle and deal decisively with impunity, injustice, bias and every action or inaction regardless of status, religion, ethnicity or creed that threatens the human rights of the citizenry on the one hand and the secularity of the Nigerian state on the other ( Olujobi & Yebisi, 2023).

Legislative Intervention, owing to the right to wear a hijab in public institutions being permissible as the status quo, the Nigerian government can consider an amendment to Section 45(1) of the 1999 Constitution (as amended) to permit public institutions through administrative policy and enactments to be able to validly limit the right to religious freedom based on the need to promote public order and safety. Such a legislative intervention would be able to adequately make obsolete the decision in Lagos State Govt. and Ors v. Asiyat Abdulkareem (Supra), as the right to religious freedom expressed by the wearing of hijabs would have been limited under the law as prescribed by Section 45 of the Constitution.

Nigeria should emulate states like Turkey, regardless of being an Islamic nation, have entrenched secular constitutions to allay any internal concerns from the international community that objective fairness devoid of religious sentiment is not being practised in the country. Indeed, state resources should be channelled towards the promotion of education, healthcare, industrialisation, and the overall wellness of the economy, and not private matters like religion. Accordingly, Nigeria must begin to dismantle the various state apparatus that is paradoxical to our projection as a secular state. For example, the dissolution of the Muslim and Christian Pilgrimage Boards where state resources are expended in sending Nigerians on pilgrimage must be considered as the same only hampers efforts at secularity.

## Conclusion

The current legal framework for the protection of human rights in Nigeria inclusive of religious rights is composed of a plethora of laws as well as institutions saddled with the responsibility of guaranteeing various human rights such as the right to wear hijab in public institutions as a religious right. This makes the role of the judiciary very critical as it is invariably responsible for giving effects to the letters of the law upon which persons who seek enforcement of their human rights can bring before it.

This article has shown that although Nigeria is not bereft of working institutions like the judiciary, and legislation to address human rights issues such as the wearing of hijabs the current legal approach without further legislative intervention or a hybrid of social and legal solutions will be inadequate.

The need for an increased political will to promote secularity in Nigeria, as reiterated in the decision in Lagos State Govt. and Ors v. Asiyat Abdulkareem (Supra *),* remains the position of the law until overturned despite the problematic consequences it occasions. As such, beyond relying on a purely legalistic approach, the Nigerian government through effecting the right policy must show that it is ready to promote peace by allaying the fears of all religious interests in the country.

The need to keep up with modern realities by channelling focus and resources towards nation-building and growth, it is submitted that religion is a private matter and should remain as such. The state should not delve into it unnecessarily especially in heterogenous societies where diverse ethnic and religious groups are in constant suspicion of each other.

## Data Availability

No data are associated with this article.
