# Supplementary material for: The legal and institutional framework for the protection of religious rights in Nigeria and the right to wear Hijab in public institutions
Source: F1000Res. 2024 Jul 3;12:537. Originally published 2023 May 23. [Version 2] doi: 10.12688/f1000research.132637.2 (PMC11184636; doi:10.12688/f1000research.132637.2)
Supplement: Peer review history

# Reviewer report: Reviewer response for version 1

**DOI:** 10.5256/f1000research.145571.r277193

**Published:** 2024-06-14

**Authors:** Chukwunye Augusta Ojeih

**Affiliations:** 1 Faculty of Law, Elizade University, Ilara-Mokin, Ondo, Nigeria

**Summary/Findings**: -

 This article discusses the constitutional and human rights provisions of Nigerians, domiciled in Nigeria, to exercise their freedom of worship/religion. However, this freedom of religion isn’t without challenges in Nigeria because of its special and peculiar kind of political, social, ethnic, and religious entities. The article notes that one of the greatest challenges in Nigeria is freedom of religion – freedom to practice one’s religious beliefs; which has led to fiery litigation in some instances.

 The wearing of hijab has engendered much debate in public institutions in Nigeria; this in itself has threatened the “supposed secular compass of the Nigerian state following the decision of the Supreme Court” in the much celebrated case of *Lagos State & Ors v Asiyat Abdulkareem* (SC/910/16) in which case judgment was tilted in favour of the plaintiff cum defendant.

 The article did justice by presenting the two sides of the divide on the matter; i.e. those for and against the wearing of hijab in public institutions.

 The comparative analysis between Nigeria and other jurisdictions is quite instructive. Other states (Nations) have placed public interests above religious sentiments/bias. The article points to the fact that although the wearing of hijab by female Muslims is a mandatory religious injunction, it should not affect the secularism of the Nigerian state. For instance in Turkey, which is a predominantly Islamic nation, the wearing of hijab in public institutions is banned. The females are allowed to wear a good head-cover but not hijab. Same goes for Tunisia, which also banned the wearing of hijab in public institutions. Worthy of note is Kazakhstan, an Islamic nation, which banned the wearing of hijab in public institutions partly for public good and because religious “extremists utilized such clothing to occasion harm to the society”.

 The article notes that reliance should not be placed exclusively on the decisions of the apex court of Nigeria alone; there is the need for legislative intervention. No religion should be promoted over and above the other in Nigeria. Every religion is good as long as it is not repugnant to natural justice, equity, and good conscience. Nigeria being a multi-faceted, multi-ethnic, and a multi-religious nation needs a balance. A healthy balance so that religious groups will not arbitrarily engage in activities which are likely to jeopardies public safety and order.

 The article recommends that Nigerians, in Nigeria, should exercise their civil liberties and religious freedom within the ambit of the law, bearing in mind overall public interest. Put simply, section 39 (1) CFRN is not absolute but circumscribed in some situations.

 The authors advocate that Nigeria should align with other nations like Turkey, Kazakhstan, and Tunisia on the issue of wearing of hijab in public institutions and the exercise of religious freedom. This is necessary for the overall wellness of the nations in terms of religious freedom and public safety.

**Relevance**: -

 The article is very significant in the area of the Nigerian constitutional law; it is good for law teachers and policy makers which will in turn affect what the journalists, as gate keepers, report.

 There is the urgent need for a healthy and balanced legislative intervention in the area of religious freedom in Nigeria, there are many deaths and destructions, especially in the Northern part of Nigeria.

 Finally, beyond the Constitutional and other statutory provisions, the right of freedom of worship is a God-given right because man is a free-moral agent. So, the issue is really balancing the provisions of section 38 (2) with section 39 (1) CFRN 1999 (as amended). What happens when a child decides to go against the religion of his/her parents/guardians?

**Strong point**: The greatest strength of the article is the call for a balanced and beneficial intervention of the legislative arm of the government.

Is the work clearly and accurately presented and does it cite the current literature?

Yes

If applicable, is the statistical analysis and its interpretation appropriate?

I cannot comment. A qualified statistician is required.

Are all the source data underlying the results available to ensure full reproducibility?

Yes

Is the study design appropriate and is the work technically sound?

Yes

Are the conclusions drawn adequately supported by the results?

Yes

Are sufficient details of methods and analysis provided to allow replication by others?

Yes

Reviewer Expertise:

Intellectual property law, Nigerian Legal system, Criminology, Law of Tort, and Equity and Trust.

I confirm that I have read this submission and believe that I have an appropriate level of expertise to confirm that it is of an acceptable scientific standard.

# Response

**Published:** 2024-06-25

**Authors:** OLUSOLA JOSHUA OLUJOBI

**Affiliations:** PUBLIC AND INTERNATIONAL LAW, Afe Babalola University, Ado Ekiti, Ekiti, Nigeria

Dear Distinguished reviewer,

 Thank you very much for investing your time and effort in reviewing our work. Your insights and feedback are greatly appreciated. May the good Lord bless you and your family abundantly.

 Warm regards,

**Dr Olujobi, Olusola**

# Reviewer report: Reviewer response for version 1

**DOI:** 10.5256/f1000research.145571.r280499

**Published:** 2024-06-12

**Authors:** Oluwatosin Kate Olanrewaju-Elufowoju

**Affiliations:** 1 Redeemer's University, Ede, Osun, Nigeria

Article Summary

 “The legal and institutional framework for the protection of religious rights in Nigeria and the right to wear Hijab in public institutions" by Caesar Manuchimso Elikwu, Olusola Joshua Olujobi, and Ebenezer Tunde Yebisi examines the intricate nature of religious rights in Nigeria, with a particular emphasis on the right to wear the hijab in public institutions. The report, which was published on May 23, 2023, emphasises the careful balance needed to protect religious freedom in a country with a multi-religious and multi-ethnic nation like Nigeria.

 The article begins by discussing the constitutional guarantee of religious rights in Nigeria and the legal limitations and interpretations of these rights. It underscores the importance of these rights being protected, while also recognizing the need for regulation to prevent conflicts. The authors review existing literature, statutes, and laws, including the 1999 Constitution of Nigeria, judicial precedents, and international conventions. A comparative analysis is made with countries such as Tunisia, Algeria, Turkey, and Kazakhstan, which have successfully managed similar issues. A key focus of the study is the Supreme Court ruling in Lagos State Govt. and Ors v. Asiyat Abdulkareem, which upheld the right of Muslim women to wear the hijab as a fundamental right. This decision has been met with mixed reactions, highlighting the ongoing debate over the extent of religious expression in public spaces. The study points out that the current legal framework in Nigeria, while adequate, requires further legislative intervention to address these issues effectively. The methodology of the research is a doctrinal legal research approach, relying on primary and secondary legal sources to provide a comprehensive analysis. The authors propose a hybrid model to mitigate the socio-legal impacts of hijab usage in public institutions. They conclude that legislative measures are necessary to align Nigeria’s legal framework with global standards and to promote societal stability.

 In conclusion, the article provides an insightful legal analysis of religious rights in Nigeria, offering practical recommendations to balance these rights with public interests. By drawing lessons from other countries, the study aims to enrich Nigeria's jurisprudence and foster a more harmonious coexistence of diverse religious practices.

 Reviewer’s Report

 The authors have done their research, thus, contributing their perspective to the body of knowledge relating to the topic of study. Their rigour of research and dexterity with literature is commendable. The literature review is robust and portrays an extensive engagement with literature.  However there are some observations which should be addressed by them as follows:

- Abstract Conclusion Page 2

The conclusion does not reflect the body of the abstract or the underlined idea of the topic. It should reflect what purpose Nigeria’s legal and institutional frameworks are adequate for? Answering this question, will help to understand what can be tweaked to reflect the topic and body of the abstract.. Perhaps, the conclusion should be reframed in line with the body of the abstract and central  idea of the topic.

- Methods Page 4

The method should give an insight into what the hybrid model is about. Spelling errors were observed in paragraph 2 of the methods, ‘...religious rights’ not ‘...religious light’; ‘...that can’ not ‘...that cans’.

- Continuation of African Charter on Human and People’s Rights (Ratification and Enforcement) Act CAP A9, LFN, 2004 on Page7

Paragraph 2, line 4 should read ‘...prohibits religious intolerance’ not ‘...religious tolerance’.

- Public Complaints Commission Page 9

The authors have not shown how the public complaints commission relates to the topic. How as the Public Complaints Commission encouraged or discouraged wearing of hijab in public institutions in Nigeria. Perhaps, a relatable example can be given.

- Truth and Reconciliation Commission Page 9

There is an omission in Line 3. ‘...between 1984 to what year”? Not 199. The authors have not related this as an institutional framework to the topic.

- The police and other security agencies Page 9

The authors should state an instance where the police and other security agencies have intervened in matters related to the wearing of hijab in public institutions. This will make this institutional framework more robust.

- Comparison with other countries - Tunisia Page 10

There is an error in line 5 ‘...resolved successfully’ not ‘...resolve successful’. In addition, the last sentence stated that “Tunisia and Turkey are two countries who have decided against its wearing in public places”. This is inconsistent with the current position in Turkey and also as portrayed in the authors’ concluding discuss on Turkey in the article.

Is the work clearly and accurately presented and does it cite the current literature?

Yes

If applicable, is the statistical analysis and its interpretation appropriate?

Not applicable

Are all the source data underlying the results available to ensure full reproducibility?

No source data required

Is the study design appropriate and is the work technically sound?

Yes

Are the conclusions drawn adequately supported by the results?

Yes

Are sufficient details of methods and analysis provided to allow replication by others?

Yes

Reviewer Expertise:

International Environmental Law, Environmental Law, Alternative Dispute Resolution, Information & Communication Law

I confirm that I have read this submission and believe that I have an appropriate level of expertise to confirm that it is of an acceptable scientific standard.

# Response

**Published:** 2024-06-25

**Authors:** OLUSOLA JOSHUA OLUJOBI

**Affiliations:** PUBLIC AND INTERNATIONAL LAW, Afe Babalola University, Ado Ekiti, Ekiti, Nigeria

**Author response to Reviewer Comments:**

 1) Abstract Conclusion Page 2: The conclusion has been revised to align closely with the abstract’s central theme, highlighting the inadequacy of Nigeria’s legal and institutional frameworks concerning the protection of religious rights and the right to wear hijab in public institutions. Therefore, reform is deemed necessary.

 2) Methods Page 4:

 The methodology section has been refined to explain the concept of a hybrid model. Spelling errors identified in paragraph 2 have been corrected: 'religious rights' replaces 'religious light', and 'that can' replaces 'that cans'.

 3) Continuation of the African Charter on Human and People’s Rights (Ratification and Enforcement) Act CAP A9, LFN, 2004 on Page 7: Corrections have been made for clarity. Paragraph 2, line 4 now reads: 'prohibits religious intolerance' instead of 'religious tolerance'.

 4) Public Complaints Commission Page 9: The authors have clarified the relevance of the Public Complaints Commission to the topic. The Commission receives and addresses complaints in the public interest, providing independent oversight to maintain and enhance public confidence. An example provided is the case of Miss Ufumaka Glory Ukpanken (Corper No. EB/A221514) v. NYSC, where the Federal High Court in Abakaliki ordered the NYSC to allow female corps members to wear skirts if it aligns with their religious beliefs, citing a violation of their fundamental rights under Section 38(1) of the 1999 Constitution.

 5) Truth and Reconciliation Commission Page 9: The authors have rectified an omission in line 3, adding 'between 1984 to 1999'. The institutional framework related to the topic has been comprehensively discussed.

 6) The police and other security agencies Page 9: The authors have provided an instance where police and other security agencies intervened in matters concerning the wearing of hijab in public institutions to strengthen the institutional framework. In Kwara State in 2019, protests erupted over the hijab policy in public schools in Ilorin, Kwara State. Police were deployed to maintain peace as tensions escalated between opposing groups. This led to heightened security measures around affected schools.

 In 2020 in Lagos State, tensions and protests also arose over the issue of hijab-wearing in public schools. Police presence was crucial in preventing clashes and ensuring compliance with government directives on hijab-wearing.

 Similarly, in Osun State, police intervened during disputes and protests regarding the enforcement of hijab policies in public schools. Their presence helped manage tensions and ensure that academic activities were not disrupted.

 These instances illustrate how police and security agencies play a pivotal role in maintaining peace and enforcing regulations related to the wearing of hijab in public institutions across Nigeria.

 7) Comparison with other countries - Tunisia Page 10: An error in line 5 has been corrected to 'resolved successfully'. Additionally, the statement regarding Tunisia and Turkey has been clarified. While Tunisia has resolved issues related to hijab-wearing, Turkey historically had restrictions on hijab in educational and government institutions, which led to protests and legal challenges. Police were involved in enforcing these regulations.

# Reviewer report: Reviewer response for version 1

**DOI:** 10.5256/f1000research.145571.r280494

**Published:** 2024-06-07

**Authors:** Olalekan Bello

**Affiliations:** 1 Leicester Law School, University of Leicester, Leicester, England, UK

**CLARITY AND SCOPE**

 This has been a refreshing paper to read in the context of the legal and institutional framework for the protection of religious rights in Nigeria and the Right to Wear Hijab in Public Institutions. The introduction provides a clear overview of the topic and its importance. The author’s goals and objectives are clear

**STRUCTURE, ORGANISATION** **AND** **WRITING STYLE**

 The paper is well-organised, with a logical flow of ideas However, the authors need to ensure that the paragraphs and sentences transition smoothly, maintaining a cohesive narrative throughout. The writing is clear and academic in tone. However, the authors are advised to watch their varying sentence structures and to employ more engaging language to enhance readability.

**LITERATURE REVIEW**

 The literature review covers key Nigerian sources but could be expanded to include more recent studies and comparative analyses from other jurisdictions facing similar issues. This would enrich the context and highlight the relevance of the Nigerian situation.

**CRITICAL ANALYSIS:**

 There is a good synthesis of existing literature and thorough engagement with the Nigerian legal frameworks detailing relevant laws and constitutional provisions. Also, case law, particularly landmark cases related to religious rights and the hijab in Nigeria has been used to strengthen the authors’ argument, providing practical insights into how laws are interpreted and enforced. However, the discussion on institutional mechanisms could be more detailed. In the comparative analysis with other jurisdictions, more detail is needed, and we need to see the value of the Algerian, Kazakhstan, and Turkish dynamics and how they can help resolve the current conundrum in Nigeria

**ORIGINALITY AND CONTRIBUTION TO KNOWLEDGE**

 The analysis provides a solid examination of the right to wear hijab in public institutions from a balanced perspective. The manuscript provides an original and comprehensive overview of the subject matter, but some sections, indicated in the intext comments, could benefit from more clarity.

**SUMMARY AND IMPLICATIONS:**

 The conclusion effectively summarises the main points but could be more explicit about the broader implications of the findings. Highlighting how these conclusions can influence policy and practice would underscore the paper’s relevance

**FUTURE RESEARCH**

 Suggesting areas for future research would be a valuable addition. Identifying gaps in the current literature and proposing specific questions for further study can guide ongoing scholarly efforts.

Is the work clearly and accurately presented and does it cite the current literature?

Yes

If applicable, is the statistical analysis and its interpretation appropriate?

Not applicable

Are all the source data underlying the results available to ensure full reproducibility?

Yes

Is the study design appropriate and is the work technically sound?

Yes

Are the conclusions drawn adequately supported by the results?

Yes

Are sufficient details of methods and analysis provided to allow replication by others?

Yes

Reviewer Expertise:

My areas of interests include Environmental Law and theory, Human Rights, Legal History, Legal Theory (Jurisprudence), Public International Law, Resource Conflicts in Frontier Economies, Third World Approaches to International Law, Law, Colonialism, and Imperialism, International law and development, Law, Security and Development, Sociolegal Research (Luhmann, Foucault and Deleuze's theories) and African legal and literary tradition

I confirm that I have read this submission and believe that I have an appropriate level of expertise to confirm that it is of an acceptable scientific standard.

# Response

**Published:** 2024-06-25

**Authors:** OLUSOLA JOSHUA OLUJOBI

**Affiliations:** PUBLIC AND INTERNATIONAL LAW, Afe Babalola University, Ado Ekiti, Ekiti, Nigeria

The authors have ensured that paragraphs and sentences transition smoothly to guarantee a cohesive narrative throughout the paper. The tone is now clear and academic. They have overhauled the varying sentence structures to ensure a more engaging language, enhancing the article's readability.

 The literature review has been expanded to include key Nigerian sources and recent studies, as well as comparative analyses from other jurisdictions facing similar issues, enriching the work and highlighting the relevance of the Nigerian context.

 Critical Analysis: The discussion on institutional mechanisms has been detailed, with a comparative analysis involving insights or lessons learned from strategies or dynamics in Algeria, Kazakhstan, and Turkey to address the current challenges in Nigeria regarding the protection of religious rights and the right to wear Hijab in public institutions.

 Originality and Contribution to Knowledge: Some sections, as indicated in the in-text comments, have been clarified to improve the article's clarity.

 The conclusion has been revised to effectively summarize the main points and explicitly discuss the broader implications of the research findings. It highlights how the study's conclusions can influence policy and practice regarding the protection of religious rights and the right to wear Hijab in public institutions, underscoring the paper’s relevance in the contemporary world.

# Reviewer report: Reviewer response for version 1

**DOI:** 10.5256/f1000research.145571.r238679

**Published:** 2024-06-03

**Authors:** Dr. Uche Nnawulezi

**Affiliations:** 1 Bowen University, Iwo, Osun, Nigeria

The article has been able to address the contentious issues bordering on the protection of religious rights in Nigeria from the perspective of the legal and institutional framework. Drawing from several questions raised and discussed by the authors, issues of religious rights and the right to wear hijab in public institutions was a controversial one given the plurality nature of Nigeria with several cultures and religious background. The authors have been able to resolve these controversies through their reliance to the statutory provisions of the law as contained in the Constitution of the Federal Republic of Nigeria, 1999 as amended. The paper, through a comparative analysis of what obtained in other jurisdictions, contributed to the resolution of the age-long controversies. The paper has contributed to knowledge and should be seen as a scholarly publication of repute. The suggestions made by the authors are really commendable and a panacea for sustainable protection of religious rights in Nigeria and beyond. In terms of relevance, the article is relevant to intellectual discourse on human rights, and has been able to resolve several controversies surrounding religious rights and the right to wear hijab in public institutions. This paper should be indexed in line with its scholarly contributions to knowledge.

Is the work clearly and accurately presented and does it cite the current literature?

Yes

If applicable, is the statistical analysis and its interpretation appropriate?

Yes

Are all the source data underlying the results available to ensure full reproducibility?

Yes

Is the study design appropriate and is the work technically sound?

Yes

Are the conclusions drawn adequately supported by the results?

Yes

Are sufficient details of methods and analysis provided to allow replication by others?

Yes

Reviewer Expertise:

International Humanitarian Law, International Human Rights Law, International Migration Law, International Criminal Law

I confirm that I have read this submission and believe that I have an appropriate level of expertise to confirm that it is of an acceptable scientific standard.

# Response

**Published:** 2024-06-21

**Authors:** OLUSOLA JOSHUA OLUJOBI

**Affiliations:** PUBLIC AND INTERNATIONAL LAW, Afe Babalola University, Ado Ekiti, Ekiti, Nigeria

Dear Distinguished Reviewer,

 Thank you for the time, effort, and dedication you invested in reviewing our scholarly work. We greatly appreciate your contributions and insights. May you continue to be enriched with wisdom and knowledge.

 Thank you once again.

 Regards,

**Dr Olujobi, Olusola**
